# Elastic thread modified minimal access cranial suspension lift for lower and middle third facial rejuvenation

**DOI:** 10.1097/MD.0000000000019381

**Published:** 2020-03-27

**Authors:** Nanze Yu, Panxi Yu, Zhifei Liu, Jeongmok Cho, Wenchao Zhang, Yutong Liu, Lin Zhu, Ang Zeng, Loubin Si, Fei Long, Yiding Xiao, Xiaojun Wang

**Affiliations:** a Department of Plastic Surgery, Peking Union Medical College Hospital, Chinese Academy of Medical Sciences and Peking Union Medical College,; b The 16th Department, Plastic Surgery Hospital, Chinese Academy of Medical Sciences and Peking Union Medical College, Beijing, China; c Banobagi Plastic Surgery Clinic, Seoul, Republic of Korea; d Chinese Academy of Medical Sciences and Peking Union Medical College, Beijing, China.

**Keywords:** elastic thread, face-lift, minimal access cranial suspension lift

## Abstract

Thread lift boasts the advantage of minimal invasion for facial rejuvenation and has been increasingly used nowadays. The purpose of this study was to evaluate the outcomes and safety of elastic thread when it was used in the modified minimal access cranial suspension (MACS) lift for rejuvenation of the lower and middle third of the face.

Forty-six patients with sagging and laxity of the lower face treated by the elastic thread modified MACS lift from December 2015 and December 2017 were enrolled in this study. A retrospective chart review was conducted. The degree of Wrinkle Severity Rating Scale (WSRS) score and satisfaction score were evaluated immediately, 6 months and 12 months after procedure. Complications during the study were also recorded.

All the patients were female, with a mean age of 50.7 ± 6.4 years and a mean follow-up period of 15.4 ± 2.1 months. The mean operation time was 114 ± 13 minutes. For the left face, the mean WSRS score was 4.0 ± 0.8 preoperatively and 3.1 ± 0.8 on the 1-year follow-up; and 4.1 ± 0.9 and 3.1 ± 0.7 on the right face (*P* < .01). Thirty-nine (84.8%) patients considered the long-term results satisfactory. There were no major complications during the follow-up period.

The elastic thread modified MACS lift is a minimally invasive, effective and safe method to improve lagging middle and lower third of the face without significant postoperative morbidity or complications.

## 1 Introduction

Face-lift, or rhytidectomy, is the workhorse of facial rejuvenation. Since the first form of face lifting was described in the early 1900s by Miller as discontinuous ellipsoidal skin excisions in natural skin creases, it has been constantly evolving from subcutaneous lift, sub-superficial musculoaponeurotic system (SMAS) lift, composite lift, separate skin and SMAS flap lift to limited lift with SMAS manipulation. Recently plastic surgeons have focused on individualized treatment plans to accommodate patient-specific facial shapes, vectors, and volumetric requirements.^[1]^

Thread lift is a minimally invasive procedure for facial rejuvenation. It satisfies patients with short operative duration, minimal scarring, rapid recovery, few complications, and thus becomes a good alternative for more invasive procedures.^[2]^ But thread lifting is not suitable for all kinds of patients, especially those who have redundant skin needing excision. Therefore, to treat patients with considerable skin sagging, we applied a new type of elastic thread (Elasticum, Korpo SRL, Genova, Italy)^[3]^ in the modified minimal access cranial suspension (MACS) lift.^[4]^ The purpose of this study was to evaluate the outcomes and safety of the combination therapy in rejuvenation of midface and upper mandibular region.

## 2 Materials and methods

### 2.1 Patients

Patients underwent lower and middle face rejuvenation with a combination therapy of elastic thread lift and modified MACS lift between December 2015 and December 2017 were enrolled in the study. Patients who had active systemic or local infections, local skin diseases that might alter wound healing, a history of psychiatric illness, obvious facial asymmetry, or soft tissue augmentation materials were excluded.

Medical records and operative records were retrospectively reviewed to evaluate postoperative outcomes and complications. The study protocol was approved by the Institutional Review Board of our hospital (S-K196) and was performed in accordance with the ethical principles of the Declaration of Helsinki. Written consent was obtained from each patient for both the surgery and publication of the results.

### 2.2 Elastic thread

**Figure 1:**
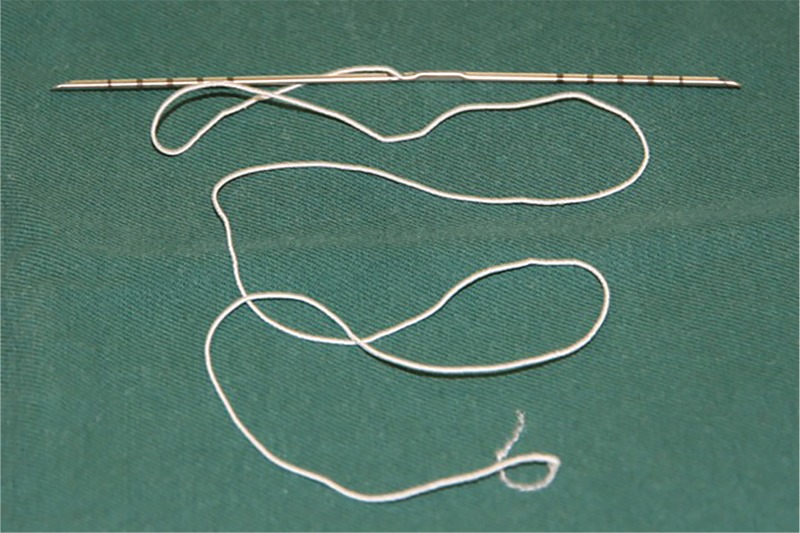
The elastic thread, Elasticum. The elastic thread is attached to the center portion of the Jano needle.

Elasticum consists of a nonabsorbable elastic thread and a two-tipped long needle (Jano needle). There are 5 scale marks of 1 cm lengths on both sides of the needle, and the elastic thread is attached to the center portion of the needle (Fig. 1). The elastic thread is made of a silicone core and a polyester sheath, which can be stretched up to twice the length of the thread itself. The two-tipped Jano needle can move forward and backward in the tissues and enable suspension and traction to be carried out without dissection.

### 2.3 Skin marking

**Figure 2:**
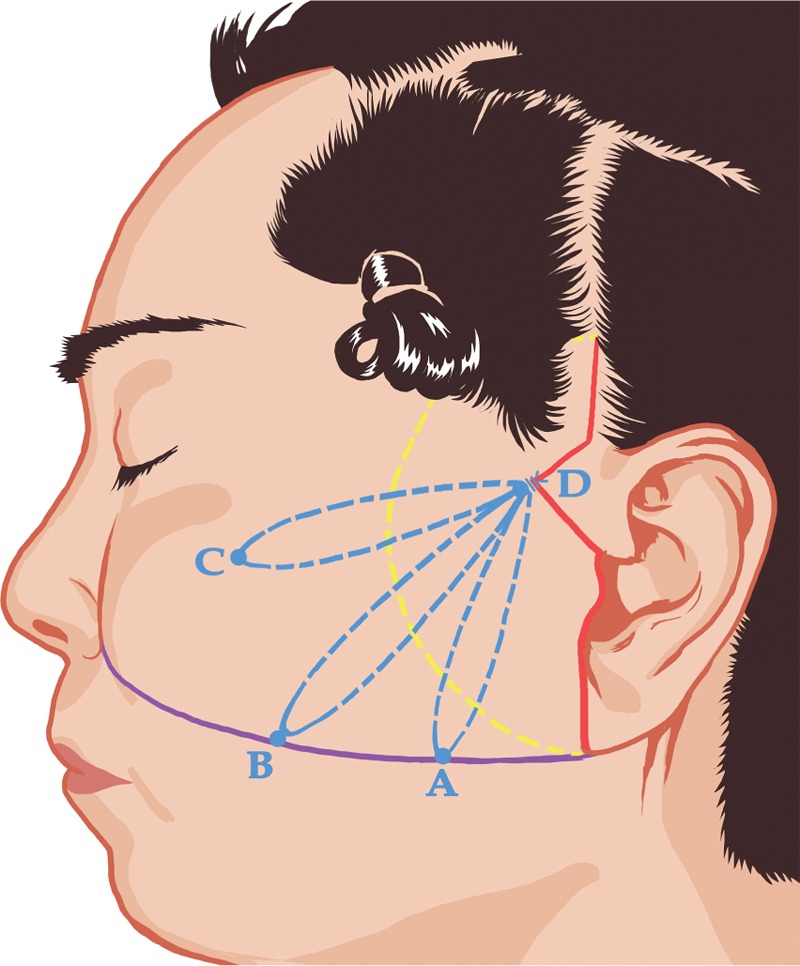
Illustration of skin anchoring points, skin incision and dissection region. On the line between the earlobe and alar base (purple line), anchoring point A is 3 cm from the earlobe, and anchoring point B is 3 cm from point B. Anchoring point C represents the malar eminence. Point D is 2 cm above the root of the zygomatic arch. The lines (blue lines) that connect points A-D, B-D and C-D represent the vectors of elastic lift. Skin incision (red line) starts at the earlobe and ascends up along the preauricular crease to reach the tragus, where it forms a triangle and then turns upward to end approximately 2 cm in the hairline. Blunt dissection is centered on the top of the tragus to form a fan-shaped region with a radius of 3 to 4 cm (yellow line).

With the patient in supine position, we firstly draw a slightly curved line from the lower pole of the earlobe to the lateral margin of the ipsilateral alar base. On this line, point A is marked 3 cm from the ear lobule, and point B is marked 6 cm from the ear lobule. Point C represents the malar eminence. Point D locates at the lower temporal region within the hairline 2 cm above the root of the zygomatic arch. The lines that connect points A-D, B-D, and C-D represent the vectors of elevation. The incision marking starts at the lower pole of the ear lobule, ascending up along the preauricular crease, to reach the tragus. Turn the incision marking forward at the top of the tragus, then angle the marking 60 degrees backward when reach the posterior hairline of the sideburn. The marking then follows the inferior temporal hairline and turns upward at the superior limit of the helix, finally ends approximately 2 cm in the hairline (Fig. 2). A small preauricular incision is made to eliminate the lobule deformity when needed.

### 2.4 Surgical technique

All procedures were performed under local anesthesia with intravenous sedation. The incision sites were infiltrated with 0.5% lidocaine and 1:100,000 epinephrine, while 0.25% lidocaine and 1:100,000 epinephrine in the undermining regions. After skin incision, a Metzenbaum scissor is used to dissect on the surface of SMAS until a full undermining is achieved in the preoperatively designed region about 3 cm in front of the incision line (Fig. 2). The Jano needle was firstly inserted at the anchor point D, down to the deep temporal fascia, then passed down toward point A in a purse-string manner with 1.5 cm long and 0.5 cm deep in the SMAS layer. It is essential to make sure a substantial part of SMAS is involved with every bite, while the facial nerve remains uninjured. The needle started to be extracted 1 cm below point A until about 0.5 cm of the tip remained in the tissue. The elastic thread was pulled through. The needle then returned back towards point D in the same manner with the posterior tip now becoming anterior to create a purse-string suture loop. The elastic thread was then pulled through at the anchoring point D. A knot was then placed securely with proper tension. The same procedures were performed between points B-D and C-D. The skin flap was re-draped without tension. The excessive skin was trimmed to accommodate the incision line. Interrupted 4–0 Vicryl buried sutures were applied to close the incision from the superior point downward. The skin of temporal region was sutured with interrupted 5–0 nylon and the preauricular incision with running 6–0 nylon. A silicone drainage was placed on each side of the face regularly and was expected to be removed within 24 hours. An aseptic compressive dressing was applied for 5 to 7 days.

### 2.5 Evaluation of outcomes

We evaluated the efficacy and safety of the procedure using several scales:

(1) the elevation degree of points A and B after surgery immediately;
(2) the WSRS score immediately, 6 months, and 12 months after surgery evaluated by three independent evaluators;
(3) satisfaction of the results was evaluated by asking patients to rate the results using Likert scales immediately, 6 months, and 12 months after surgery;
(4) complications during the study were also recorded.

### 2.6 Statistical analysis

Statistical analyses were performed using SPSS version 20.0 (Chicago, IL). The paired *t* test, Wilcoxon signed rank test were used to compare the scores before and after surgery. A *P* value < .05 was considered statistically significant.

## 3 Results

Among all the 52 female patients underwent lower and middle face rejuvenation with elastic thread modified MACS lift between January 2015 and December 2017, 46 patients observed and followed up for more than 1 year were enrolled in the study. The mean age of the patients was 50.7 ± 6.4 years (mean ± SD, range, 40–65 years), with a mean body mass index (BMI) of 25.1 ± 2.3 kg/m^2^ (mean ± SD, range, 20.1–31.5 kg/m^2^), and their mean follow-up period was 15.4 ± 2.1 months (mean ± SD, range, 12–18 months).

All patients underwent operative treatment performed by the same surgery group. The mean operation time was 114 ± 13 minutes (range, 91–134 minutes). Five (10.9%) patients presented with mild swelling of the surgical site, which was corrected by slight compression therapy. Seroma was discovered in 3 (6.5%) patients and was treated with aspiration and prolonged compressive dressing. There were no major complications requesting debridement, re-operation or removal of the thread during the follow-up period. No obvious facial asymmetry was found.

The elevation of points A and B were measured individually. On the right side, the elevation was 2.4 ± 0.4 cm for point A and 1.6 ± 0.6 cm for point B. On the left side, the elevation was 2.5 ± 0.3 cm for point A and 1.8 ± 0.5 cm for point B. Points A and B showed remarkable lifting effect on both sides.

**Figure 3:**
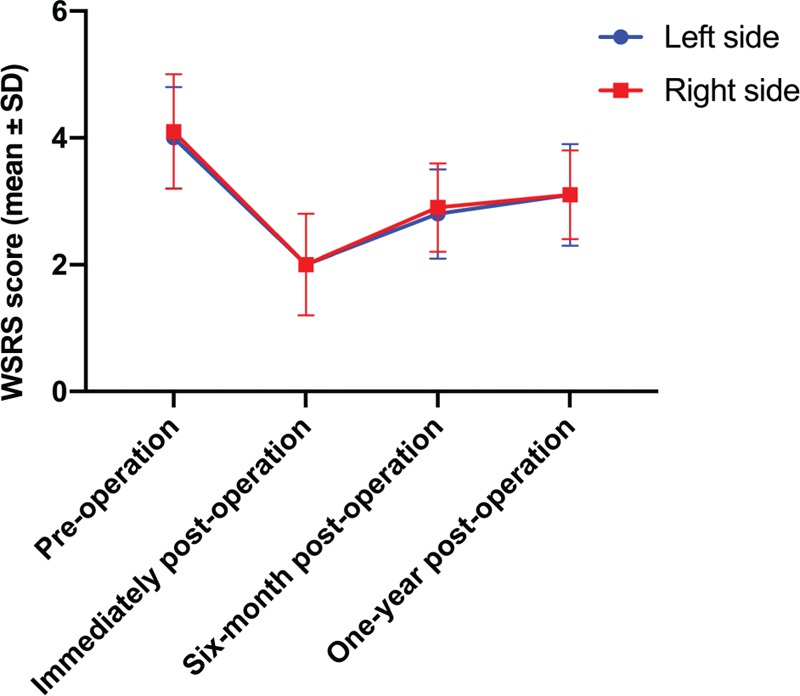
Changes of Wrinkle Severity Rating Scale (WSRS) score. Comparing to the preoperative condition, immediate and long-term improvement of WSRS scores were statistically significant (*P* < .05).

Pre- and post-operative photographs were independently shown to three plastic surgery staff blinded to the procedure. Those evaluators were asked to determine the WSRS score for each patient. For the left face, the WSRS score was 4.0 ± 0.8 (mean ± SD) for preoperative baseline, 2.0 ± 0.8 immediately after operation, 2.8 ± 0.7 on 6-month follow-up and 3.1 ± 0.8 on 1-year follow-up. For the right face, the WSRS score was 4.1 ± 0.9 for preoperative baseline, 2.0 ± 0.8 immediately after operation, 2.9 ± 0.7 on 6-month follow-up and 3.1 ± 0.7 on 1-year follow-up. The degree of improvement, when compared to preoperative baseline, was statistically significant. (Fig. 3)

**Figure 4:**
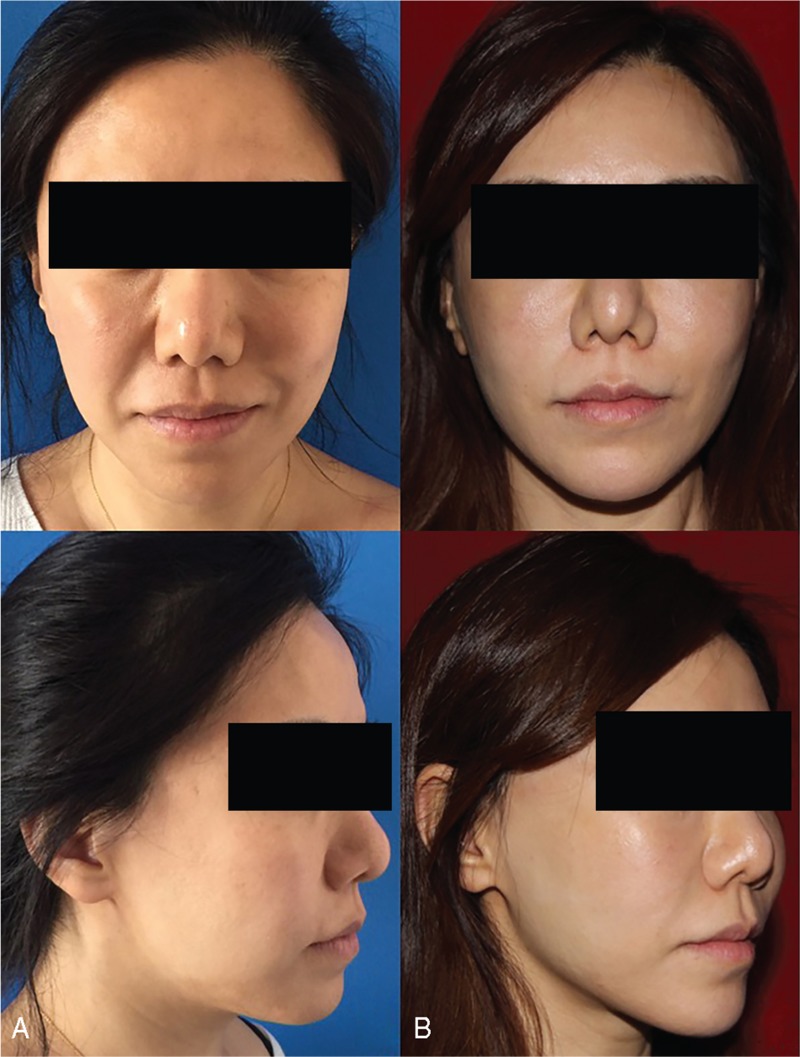
A 45-year-old female patient with moderate skin sagging and wrinkling. (A) Preoperative frontal and oblique views. (B) One-year postoperative frontal and oblique views. Notice the stable improvement of the lower and middle third of her face.

**Figure 5:**
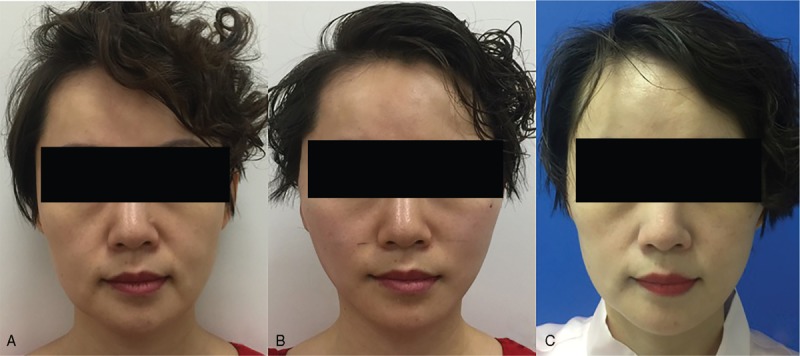
A 42-year-old female patient with moderate skin sagging and wrinkling. (A) Preoperative frontal view. (B) Immediate postoperative frontal view. (C) One-year postoperative frontal view. Her lower facial contour and deep nasolabial folds were significantly improved.

Patient-reported satisfaction rate was evaluated using Likert scales with scales of excellent, very good, good, fair and poor. Forty-five (97.8%) patients were satisfied with the result immediately after the procedure. At 6-month and 1-year follow-ups, respectively 41 (89.1%) and 39 (84.8%) patients considered the results satisfactory (Figs. 4 and 5).

## 4 Discussion

Plastic surgery patients often wish to disguise the effects of age, and the demand for a more effective, less time-consuming, and longer-lasting method for facial rejuvenation has increased year after year.^[5]^ Since Sulamanidze^[6]^ introduced a minimally invasive procedure for lifting and rejuvenating facial tissues using bidirectional barbed sutures, various minimally invasive facial rejuvenation procedures, such as Woffles thread lifting,^[7]^ Isse's endo progressive lifting,^[8]^ and silhouette lifting,^[9]^ have been developed. Therefore, thread lifts became popular and replaced, to some extent, the conventional face-lift methods.^[10]^ The elastic thread is a kind of nonabsorbable suture material sheathed with braided polyester. It is reported that the collagen matrix can infiltrate into the interstices of individual filaments, therefore, to endow the braided suture the characteristics of a ligament.^[11]^ Instead of pulling and suspending, the elastic lift repositions soft tissues in a more natural way using the elasticity of silicone. Like other thread lift methods, the effect of elastic lift is limited in patients with redundant skin, in which condition, other lift techniques must be performed to remove the excessive skin.

MACS lift, first described by Tonnard and Verpaele,^[12]^ is a short scar face lift with purse-string sutures in various locations in the SMAS, suspending ptotic facial tissues to the deep temporal fascia above the arch. This procedure reduces recovery time and morbidity, and the results seem to be as stable as traditional face lift techniques.^[4]^ Thus, it has quickly become one of the more popular face lift procedures performed all around the world.^[1]^

In this study, we applied elastic thread in the modified MACS lift to improve the lower facial contour and deep nasolabial folds. We adopted the basic concept of MACS lift during our procedure. When marking the surgical incision, instead of a zigzag pattern 2 mm within the anterior and lower border of the sideburn, we designed an equilateral triangle posterior to the sideburn (Fig. 2). This triangular broken line prolongs the incision, broadens the operative space, facilitated manipulations, and further secures surgical safety and enhances efficiency. Additionally, the extension into the temporal region perfectly hides the postoperative scar in the hairline, and at the meantime permits more elimination of excess temporal skin.

Although extensive dissection and SMAS flap elevation may produce longer-lasting effects, they are closely related to prolonged recovery time and higher complication rate. In the study, the dissection range is even 1 to 2 cm smaller than MACS lift. It is centered on the top of the tragus to form a fan-shaped peeling region with a radius of 3 to 4 cm (Fig. 2). The layer of dissection is also important. Dissection in the temporal region should be carried out beneath the superficial temporal fascia to avoid hair follicle damage and provide sufficient tension of subcutaneous sutures. Cautions should always be taken to leave some fat on the bottom of the flap to avoid puckering deformity. Facial nerve injury is one of the most severe complications occurring in facial lift procedure and is related to incorrect dissection layer. Around the external canthus, a superficial dissection at the subcutaneous layer ensures the integrity of the frontal and zygomatic branches, while on the masseter a careful dissection on the surface of SMAS reduces the risk of intra-parotid facial nerve branch injury. Since the dissection is limited within the anterior margin of the masseter, more distal branches of facial nerve are kept intact. A complete release between the skin and the orbicularis oculi must be performed to eliminate crow's feet.

The limited dissection and insufficient skin elevation are fully made up by the suspension of elastic threads. The nonabsorbable threads substantially incorporated into the SMAS to guarantee a durable result. The vertical vector elevates the lower face, and meanwhile repositions the severely sagging soft tissue. Line B-D improves the aging appearance of the nasolabial folds. Line C-D supports the infraorbital region by lifting the malar pad, thus to achieve mid-facial rejuvenation. Purse-string sutures in the SMAS layer produce substantial anchoring and create multiple microimbrications, which restores tissue volume in the suspension direction and reshapes facial contour after knots are placed with moderate tension.

Regarding to the safety and complication of this technique, we encountered some postoperative pain, mild swelling and seromas, most of which are self-limited and can be relieved by proper intervention. All the patients recovered well with a short convalescence. No severe complications, such as nerve injury, infection, hematoma, skin necrosis or hair loss were detected.

## 5 Conclusion

Our study showed that the elastic thread modified MACS lift is a minimally invasive, effective and safe method to rejuvenate middle and lower third of the face without significant postoperative morbidity or complications. A high level of patient satisfaction is also achieved.

## Author contributions

**Conceptualization:** Nanze Yu, Panxi Yu, Zhifei Liu, Jeongmok Cho.

**Data curation:** Nanze Yu, Panxi Yu, Wenchao Zhang, Yutong Liu.

**Formal analysis:** Lin Zhu, Ang Zeng, Loubin Si, Fei Long, Yiding Xiao.

**Investigation:** Nanze Yu, Panxi Yu, Zhifei Liu, Jeongmok Cho.

**Methodology:** Nanze Yu, Panxi Yu Zhifei Liu, Jeongmok Cho, Wenchao Zhang, Yutong Liu, Lin Zhu, Ang Zeng, Loubin Si, Fei Long, Yiding Xiao, Xiaojun Wang.

**Project administration:** Nanze Yu, Panxi Yu, Zhifei Liu.

**Resources:** Zhifei Liu, Jeongmok Cho.

**Software:** Nanze Yu, Panxi Yu.

**Supervision:** Zhifei Liu, Jeongmok Cho.

**Writing – original draft:** Nanze Yu, Panxi Yu.

**Writing – review & editing:** Nanze Yu, Panxi Yu Zhifei Liu, Jeongmok Cho.
